# Neutrophil-to-lymphocyte ratio, monocyte-to-lymphocyte ratio, and platelet-to-lymphocyte ratio in people with bipolar disorder: A systematic review and meta-analysis

**DOI:** 10.1192/j.eurpsy.2026.10226

**Published:** 2026-07-31

**Authors:** D. Cavaleri, G. Cucchi, M. Citton, M. Monti, C. Crocamo, G. Carrà, F. Bartoli

**Affiliations:** Università degli Studi di Milano-Bicocca , Monza, Italy

## Abstract

**Introduction:**

Immune-inflammatory dysregulation has gained increasing attention among the possible causal frameworks of bipolar disorder (BD) and its relapsing-remitting course. In particular, inflammatory indices derived from the complete blood count (CBC) – including the neutrophil-to-lymphocyte ratio (NLR), the monocyte-to-lymphocyte ratio (MLR), and the platelet-to-lymphocyte ratio (PLR) – have been studied as a proxy for immune-inflammatory processes.

**Objectives:**

The aim of this systematic review and meta-analysis is to provide a better understanding of the role of inflammation in BD and to identify accessible markers that could inform clinical management.

**Methods:**

This work was conducted in accordance with the Meta-analyses Of Observational Studies in Epidemiology (MOOSE) Reporting Guidelines. A protocol was registered in Open Science Framework (OSF) Registries on June 23, 2025 [doi: 10.17605/OSF.IO/NPCX6].

**Results:**

After a full-text review, 51 studies, based on 49 independent samples, met eligibility criteria and were included. Subjects with BD showed higher NLR (k=35; SMD=0.44, 95%CI: 0.30 to 0.59, p<0.001; I2=92.2%) and MLR (k=20; SMD=0.28, 95%CI: 0.14 to 0.42, p<0.001; I2=88.1%) than healthy controls (HCs), while no significant differences in PLR were observed (k=29; SMD=0.05, 95%CI: –0.06 to 0.16, p=0.373; I2=85.5%). In subjects with a manic episode, NLR (k=24; SMD=0.62, 95%CI: 0.41 to 0.83, p<0.001; I2=93.7%), MLR (k=14; SMD=0.51, 95%CI: 0.37 to 0.65, p<0.001; I2=78.3%), and PLR (k=21; SMD=0.18, 95%CI: 0.04 to 0.33, p=0.014; I2=85.8%) were higher than in HCs. In subjects with a depressive episode, PLR was significantly lower than in HCs (k=10; SMD=–0.14, 95%CI: –0.22 to –0.06, p<0.001; I2=27.2%), and no significant differences emerged for NLR (k=12; SMD=0.19, 95%CI: –0.03 to 0.42, p=0.091; I2=91.2%) and MLR (k=8; SMD=0.11, 95%CI: –0.11 to 0.32, p=0.342; I2=88.5%). In euthymic individuals, NLR was higher than in HCs (k=15; SMD=0.37, 95%CI: 0.14 to 0.61, p=0.002; I2=86.8%), while no significant differences were found in MLR (k=8; SMD=0.11, 95%CI: –0.19 to 0.42, p=0.459; I2=85.9%) and PLR (k=13; SMD=–0.03, 95%CI: –0.23 to 0.16, p=0.756; I2=77.3%).

**Conclusions:**

Despite generally high statistical heterogeneity, this work strengthens the case for higher inflammation in people with BD compared to HCs. In particular, mania bears the most marked inflammatory profile. The presence of residual, trait-like inflammation in euthymia was also observed. Taken together, our findings support the role of NLR, MLR, and PLR as accessible trait and state markers of BD.

**Disclosure of Interest:**

None Declared

